# Detection of early adenocarcinoma of the esophagogastric junction by spraying an enzyme-activatable fluorescent probe targeting Dipeptidyl peptidase-IV

**DOI:** 10.1186/s12885-020-6537-9

**Published:** 2020-01-28

**Authors:** Keiko Yamamoto, Shunsuke Ohnishi, Takeshi Mizushima, Junichi Kodaira, Masayoshi Ono, Yutaka Hatanaka, Kanako C. Hatanaka, Yugo Kuriki, Mako Kamiya, Nobuyuki Ehira, Keisuke Shinada, Hiroaki Takahashi, Yuichi Shimizu, Yasuteru Urano, Naoya Sakamoto

**Affiliations:** 1 Division of Endoscopy, Hokkaido University Hospital, N14, W5, Kita-ku, Sapporo, 060-8648 Japan; 2 Department of Gastroenterology and Hepatology, Hokkaido University Graduate School of Medicine, N15, W7, Kita-ku, Sapporo, 060-8638 Japan; 3 Department of Gastroenterology, Japanese Red Cross Kitami Hospital, N6, E2, Kitami, 090-8666 Japan; 4 Department of Gastroenterology, Keiyukai Daini Hospital, N3-7-1, Hondori, Shiroishi-ku, Sapporo, 003-0027 Japan; 5 Department of Gastroenterology, Hakodate Municipal Hospital, 10-1, Minato-cho 1, Hakodate, 041-8680 Japan; 6 Department of Surgical Pathology, Hokkaido University Hospital, N14, W5, Kita-ku, Sapporo, 060-8648 Japan; 7 Laboratory of Chemical Biology and Molecular Imaging, Graduate School of Medicine, University of Tokyo, 7-3-1 Hongo, Bunkyo-ku, Tokyo, 113-0033 Japan; 8 Department of Gastroenterology, Keiwakai Ebetsu Hospital, Ebetsu, 81-81-6, Yoyogi-cho, Ebetsu, 069-0817 Japan; 9 Japan Agency for Medical Research and Development (AMED)-CREST, 7-1 Ootemachi-1, Chiyoda-ku, Tokyo, 100-0004 Japan

**Keywords:** Fluorescence imaging, Dipeptidyl peptidase-IV, Adenocarcinoma of the esophagogastric junction

## Abstract

**Background:**

It is still difficult to detect and diagnose early adenocarcinoma of the esophagogastric junction (EGJ) using conventional endoscopy or image-enhanced endoscopy. A glutamylprolyl hydroxymethyl rhodamine green (EP-HMRG) fluorescent probe that can be enzymatically activated to become fluorescent after the cleavage of a dipeptidyl peptidase (DPP)-IV-specific sequence has been developed and is reported to be useful for the detection of squamous cell carcinoma of the head and neck, and esophagus; however, there is a lack of studies that focuses on detecting EGJ adenocarcinoma by fluorescence molecular imaging. Therefore, we investigated the visualization of early EGJ adenocarcinoma by applying EP-HMRG and using clinical samples resected by endoscopic submucosal dissection (ESD).

**Methods:**

Fluorescence imaging with EP-HMRG was performed in 21 clinical samples resected by ESD, and the fluorescence intensity of the tumor and non-tumor regions of interest was prospectively measured. Immunohistochemistry was also performed to determine the expression of DPP-IV.

**Results:**

Fluorescence imaging of the clinical samples showed that the tumor lesions were visualized within a few minutes after the application of EP-HMRG, with a sensitivity, specificity, and accuracy of 85.7, 85.7, and 85.7%, respectively. However, tumors with a background of intestinal metaplasia did not have a sufficient contrast-to-background ratio since complete intestinal metaplasia also expresses DPP-IV. Immunohistochemistry measurements revealed that all fluorescent tumor lesions expressed DPP-IV.

**Conclusions:**

Fluorescence imaging with EP-HMRG could be useful for the detection of early EGJ adenocarcinoma lesions that do not have a background of intestinal metaplasia.

## Background

Adenocarcinoma of the esophagogastric junction (EGJ) is a cancer that develops in the transition zone between the esophagus and the stomach, and its incidence has rapidly increased in recent decades, especially in Western countries [1]. Even in Asia, including Japan, there is concern that the incidence of esophagogastric junctional adenocarcinoma will increase following a decrease in the *H. pylori* infection rate [2, 3]. Early stage adenocarcinoma of the EGJ can be endoscopically resected, such as by endoscopic submucosal dissection (ESD), and is expected to achieve good oncological outcomes [4–6]. On the other hand, the prognosis of advanced esophageal adenocarcinoma, including esophagogastric adenocarcinoma and Barrett’s adenocarcinoma, is poor, and its early detection is necessary for better prognosis [7–9]. Several modalities can detect early carcinoma, such as narrow band imaging (NBI), high magnification chromoendoscopy, indigo carmine dye spraying, and acetic acid spraying [10]. However, detecting early EGJ adenocarcinoma is still difficult because of the following reasons: (1) the tumor develops at a region of physiological construction; (2) the tumor often has a macroscopically flat morphology and exhibits a microsurface structure similar to that of the surrounding non-tumor region; and (3) the tumor sometimes extends under the squamous epithelium [11]. Therefore, the development of a novel and simple method to detect early EGJ adenocarcinoma is needed.

Dipeptidyl peptidase-IV (DPP-IV) is a prolyl-specific protease expressed on the cell surface of a variety of tissues, particularly in the kidney and colon [12]. Certain human cancers, such as those of the prostate, thyroid, and esophagus, show the overexpression of DPP-IV [13–17]. It has recently been reported that glutamylprolyl hydroxymethyl rhodamine green (EP-HMRG), a fluorescent targeting agent based on the fluorophore rhodamine green, becomes fluorescent after cleavage of a DPP-IV-specific sequence, it can be activated within several minutes by topical application in cases of esophageal cancer, and its sensitivity and specificity for diagnosis are comparable to those of iodine chromoendoscopy [18]. In addition, we recently demonstrated that EP-HMRG was useful for the rapid detection of superficial head and neck cancer [19]. However, it remains to be elucidated whether EP-HMRG can be applicable as a fluorescent targeting agent in the detection of early EGJ adenocarcinoma. We therefore evaluated whether early EGJ adenocarcinoma could be detected by the application of EP-HMRG with the use of fresh clinical samples obtained by ESD.

## Methods

### Definition of carcinoma at the EGJ and Barrett’s esophagus

We defined cancer at the EGJ according to the Japanese classification [20]. In this classification, the area extending 2 cm above to 2 cm below the EGJ is designated as the EGJ area. Tumors having their epicenter in this area are designated as EGJ carcinomas irrespective of histological type. The location of an EGJ carcinoma is described using the symbols E (proximal 2 cm segment) and G (distal 2 cm segment), with the dominant area of invasion described first; i.e., E, EG, E = G (both areas equally involved), GE, or G. Barrett’s epithelium was endoscopically diagnosed when columnar epithelium was continuously observed from the stomach to the distal side of the esophagus. In the United States of America and most European countries, the diagnosis of Barrett’s epithelium requires histologically confirmed intestinal metaplasia [21, 22]. However, in England and Asian countries, including Japan, histological demonstration of goblet cell is not required [23–26]. In this study, we defined Barrett’s epithelium as the continuous columnar epithelium from the stomach with or without intestinal metaplasia. The presence of circular Barrett mucosa extending longitudinally for 3 cm or more in length was classified as long segment Barrett esophagus (LSBE). On the other hand, the presence of circular Barrett mucosa less than 3 cm in length or the presence of non-circular Barrett mucosa was designated as short segment Barrett esophagus (SSBE) [26].

### Enzyme-activatable fluorescent targeting agent

The EP-HMRG probe was purchased from Goryo Chemical (Sapporo, Japan), resuspended in 10 mM dimethyl sulfoxide (Sigma-Aldrich, St. Louis, MO, USA) and then stored at − 80 °C. Before use, the EP-HMRG suspension was thawed to room temperature and diluted to 100 μM with phosphate-buffered saline (PBS, Life Technologies, Carlsbad, CA, USA).

### Patients

This study prospectively reviewed early EGJ adenocarcinoma resected by ESD in 23 consecutive patients at five hospitals between May, 2016 and June, 2018. All ESD procedures were performed by experienced endoscopists. We included 21 cases that met the definition of EGJ carcinoma, and excluded the cases in which complete en bloc resection was not performed, as well as cases whose specimens were too damaged for histological investigation.

The resected specimen was immediately extended on a black rubber mat and fixed with pins, and then 100 μM EP-HMRG was sprayed onto the specimen. Fluorescence imaging was performed using a handheld fluorescence imaging system (Discovery; INDEC Medical Systems, Santa Clara, CA, USA) that captured white-light images and fluorescence images with 450–490 nm blue excitation light. The fluorescence images were recorded every minute for 10 min after the EP-HMRG administration. Subsequently, the specimens were washed with PBS and observed using an endoscope (H290Z, Olympus, Tokyo, Japan) under white light.

The fluorescence intensities were measured with ImageJ software (National Institutes of Health, Rockville, MD, USA). We set regions of interest (ROIs) at the area with the most fluorescence signal in the tumor lesion and in the non-tumor region adjacent to the tumor lesion. The mean fluorescence intensity of each ROI was measured as pixel intensity values ranging from 0 to 255, and the contrast-to-background ratio (CBR) was also measured.

### Ethics statement

The Ethical Review Committee of each hospital approved this ex vivo clinical study protocol. All patients provided informed consent to participate in this study.

### Pathological examination

Specimens were fixed in 40 g/L formaldehyde saline, embedded in paraffin, and cut into 5-μm sections. Tissue sections were stained with hematoxylin and eosin and then microscopically examined for the histological type, tumor size, depth of invasion, lymphovascular invasion, and resected margin by an experienced pathologist (KCH), according to the World Health Organization classification. Immunohistochemical analysis of DPP-IV expression was performed using an anti-DPP-IV antibody (Novus Biologicals, Littleton, CO, USA). The subtype of intestinal metaplasia in the non-tumor region was determined using the MUC5AC (Agilent, Santa Clara, CA, USA), MUC6 (Abcam, Cambridge, UK), MUC2 (Spring Bioscience, Pleasanton, CA, USA), and CD10 (Agilent) expression patterns. MUC5AC and MUC6 are markers of the gastric phenotype, whereas MUC2 and CD10 are markers of the intestinal phenotype. We defined the complete type as decreased expression of gastric mucin (MUC5AC or MUC6) and co-expression of MUC2 and CD10. The incomplete type was defined as the expression of MUC5AC or MUC6 and MUC2 [27].

### Statistical analysis

Receiver operating characteristic (ROC) curves were used to determine the sensitivity, specificity, and accuracy. All analyses were performed using GraphPad Prism version 6 (GraphPad Software, San Diego, CA, USA).

## Results

### Patient characteristics

We included 21 patients with 21 lesions (Table 1). Four lesions were located at the E, six lesions at the EG, two lesions at the E = G, four lesions at the GE, and five lesions at the G, according to the Japanese classification of gastric adenocarcinoma [20]. One lesion developed at long-segment Barrett’s esophagus (LSBE, case #7). In four cases, the cancer lesion was surrounded by complete or incomplete intestinal metaplasia. One lesion developed after radiotherapy (case #4). Thirteen lesions were < 20 mm in size, and 19 lesions had differentiated type as the main histological type.

**Table 1:** Patient characteristics

No.	Age /Sex	Loca -tion	Non-tumor region adjacent to tumor		Size (mm)	Macroscopic type	Histological type	Depth	Expression of DPP-IV Tumor /background	CBR at 10 min	PT (min)
			Mucosa	Main type of IM							
1	70s/M	GE	SSBE	–	23	Is + IIa + IIc	tub1 > 2	MM	+/−	6.4	63
2	60s/F	G	Gastric mucosa	complete	12	IIc	por2 > tub2	SM	+/+	0.5	11
3	50s/M	E	SSBE	–	15	IIa	tub1	SMM	+/−	2.3	74
4	60s/M	E	SSBE	–	10	IIc + IIa	tub1	MM	−/−	1.1	33
5	70s/M	G	Gastric mucosa	complete	28	IIa + IIc	tub1	SM	+/+	1.3	80
6	70s/M	E	Squamous epithelium	–	17	IIb	tub1	DMM	+/−	2.1	84
7	50s/M	E	LSBE	income -plete	7	IIc	tub1	MM	+/+	2.4	25
8	80s/M	E = G	SSBE	–	21	IIc	tub2	DMM	+/−	3.6	67
9	60s/F	GE	SSBE	–	20	IIa	tub1	M	+/−	2.2	70
10	70s/F	E = G	Gastric mucosa	–	23	IIc	muc > tub1	SM	+/−	2.9	27
11	60s/M	EG	Gastric mucosa	complete	8	IIa	tub1 > 2	SMM	+/+	1.2	36
12	80s/M	EG	SSBE	–	25	IIc	tub1	SM	+/−	6.0	41
13	50s/M	GE	Gastric mucosa	–	9	IIc	tub1	M	+/−	2.2	25
14	70s/M	G	Gastric mucosa	–	10	I	tub1 > pap	M	+/−	0.9	31
15	60s/M	EG	SSBE	–	48	IIc + IIa	pap	M	+/−	12.6	186
16	80s/M	EG	Squamous epithelium	–	20	IIa	pap	M	+/−	2.7	36
17	40s/M	G	Gastric mucosa	–	28	IIa + IIc	tub2 > 1	SM	+/−	2.9	53
18	60s/M	EG	Squamous epithelium	–	19	IIc	tub1	DMM	+/−	2.0	41
19	70s/M	GE	Gastric mucosa	–	10	IIa	tub1 > 2	MM	+/−	2.9	23
20	80s/F	G	Gastric mucosa	–	12	IIa	tub1	M	+/−	2.2	52
21	50s/M	EG	Squamous epithelium	–	25	IIa + IIc	tub1	DMM	+/−	4.7	40

Tumor location was determined according to the Japanese classification of gastric carcinoma [20]. *CBR* Contrast-to-background ratio, *SSBE* Short-segment Barrett’s esophagus, *LSBE* Long-segment Barrett’s esophagus, *IM* Intestinal metaplasia, *MM* Muscularis mucosae, *SMM* Superficial muscularis mucosae, *DMM* Deep muscularis mucosae, *M* Mucosa, *SM* Submucosa, *tub* Tubular adenocarcinoma, *pap* Papillary adenocarcinoma, *por* Poorly differentiated adenocarcinoma, *PT* Procedure time

### Fluorescence imaging of early EGJ adenocarcinoma

**Fig. 1:**
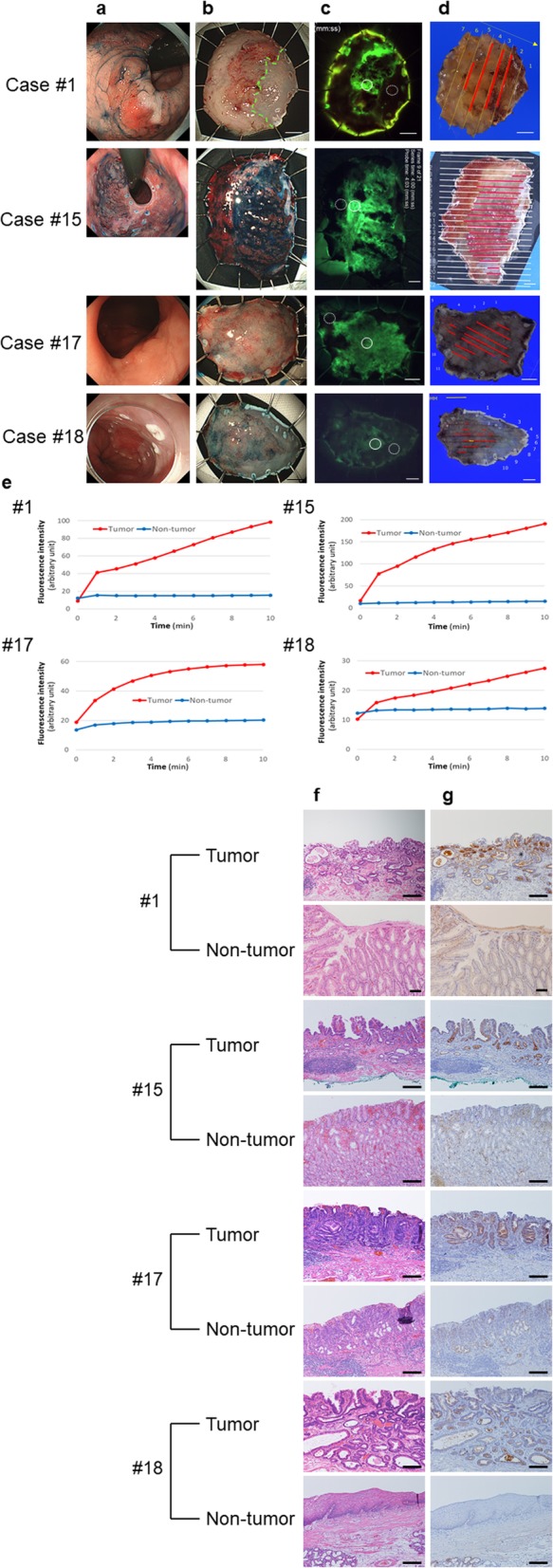
Fluorescence imaging with EP-HMRG of the four representative cases (cases #1, #15, #17, and #18). **a** Endoscopic imaging with white light before endoscopic submucosal dissection (ESD). **b** Endoscopic imaging with white light after ESD. The squamocolumnar junction (SCJ) is shown as a green line (case #1). **c** Fluorescence imaging after EP-HMRG spraying. The ROI of the tumor is the solid circle, while the ROI of non-tumor is the dotted circle. **d** Resected specimen mapping for the tumor region. The adenocarcinoma is shown as red lines. **e** Time course of the fluorescence intensity of the tumor lesion and non-tumor region after spraying EP-HMRG. **f** Hematoxylin and eosin staining and immunohistochemical examination investigating DPP-IV expression in the tumor and non-tumor regions. **g** Immunohistochemical examination investigating DPP-IV expression in the tumor and non-tumor regions. Scale bars of **b**–**d**, 5 mm. Scale bars of **f** and **g**, 200 μm

The resected specimens were sprayed with EP-HMRG, and fluorescence images were obtained every minute for 10 min. The CBRs 10 min after spraying EP-HMRG are shown in Table 1. Sixteen of the 21 lesions had sufficient CBRs (≥2). Four representative cases (cases #1, #15, #17 and #18) are shown in Fig. 1. Tumor lesions, but not the non-tumor region, became fluorescent immediately after spraying EP-HMRG (Fig. 1c). Histological mapping confirmed that the fluorescent region was almost identical to the cancer lesion (Fig. 1c and d). The fluorescence intensity in the tumor lesions showed a time-dependent increase (Fig. 1e). All representative cases showed sufficient CBRs within a few minutes after EP-HMRG spraying (Table 1). The pathological examination revealed that the tumor lesions expressed DPP-IV, but the non-tumor region did not express DPP-IV (Fig. 1f and g).

### Fluorescence imaging of the tumor extending under the squamous epithelium

**Fig. 2:**
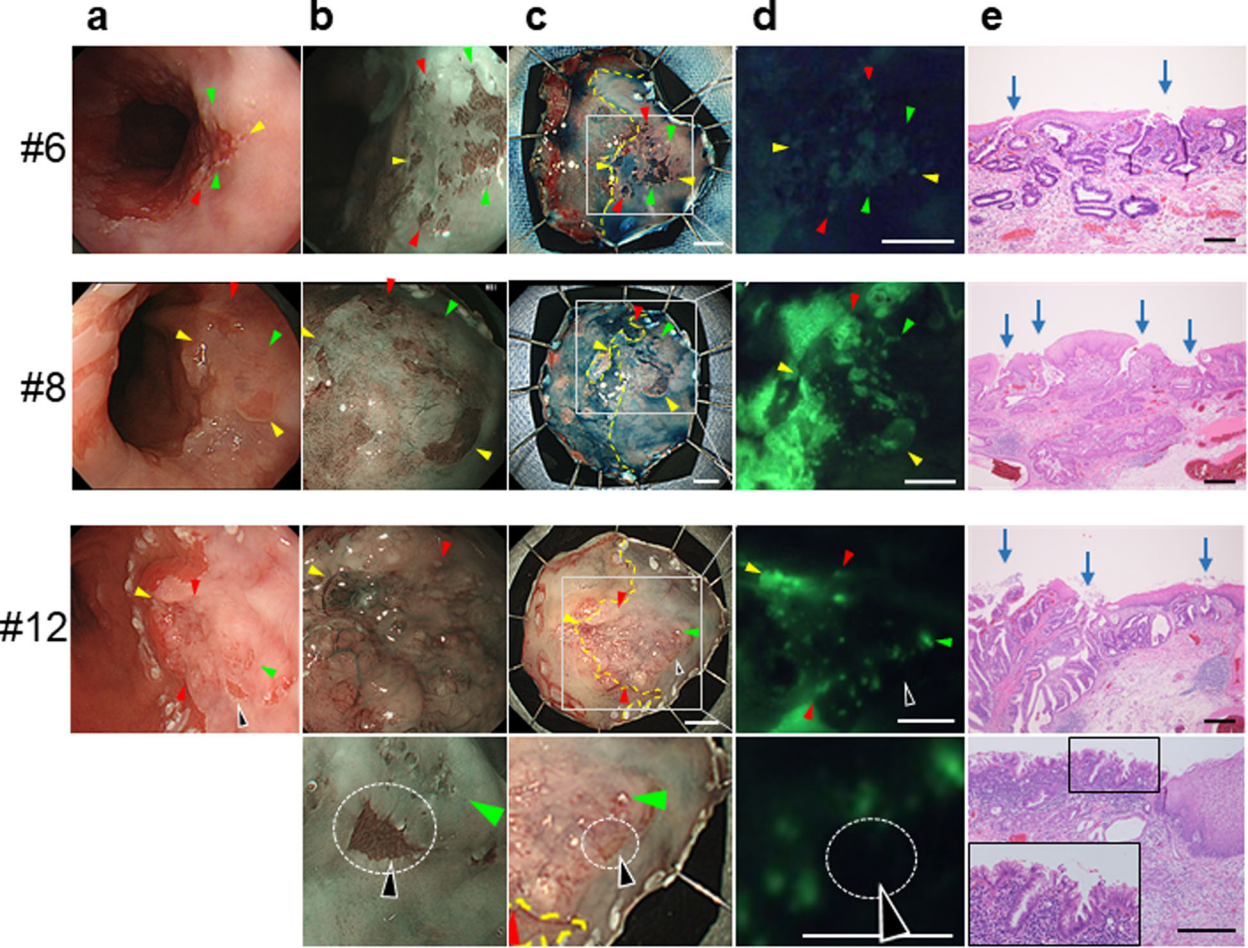
Fluorescence imaging with EP-HMRG of the tumor opening on the squamous epithelium. **a** Endoscopic imaging with white light before endoscopic submucosal dissection (ESD). **b** Magnifying narrow band imaging (M-NBI). **c** Endoscopic imaging with white light after ESD. The squamocolumnar junction (SCJ) is shown as a yellow line. **d** Fluorescence imaging after EP-HMRG spraying. **e** Pathological feature of the lesion extending under the squamous epithelium (hematoxylin and eosin staining). Arrows indicate the tumor opening on the squamous epithelium. Scale bars of (**c** and **d**), 5 mm. Scale bars of (**e**), 200 μm. The arrowheads of each color in (**a**–**d**) correspond to those of the same color in each case. The dotted circles indicate columnar epithelium without atypia (case #12)

Notably, in several cases (cases #6, #8, and #12), the tumor extended under the squamous epithelium, and most areas of the tumor tissue were covered. However conventional endoscopy and NBI showed several point-like structures or small white spots in the squamous epithelium (Fig. 2a and b), and these structures became fluorescent after spraying EP-HMRG (Fig. 2d). The pathological examination showed that they were all tumor glands that arised toward the surface and opened in the squamous epithelium (Fig. 2e). The area completely covered by squamous epithelium did not become fluorescent. Columnar epithelial islands without atypia also did not emit fluorescence (Fig. 2, case #12).

### Fluorescence imaging of early EGJ adenocarcinoma with a background of intestinal metaplasia

**Fig. 3:**
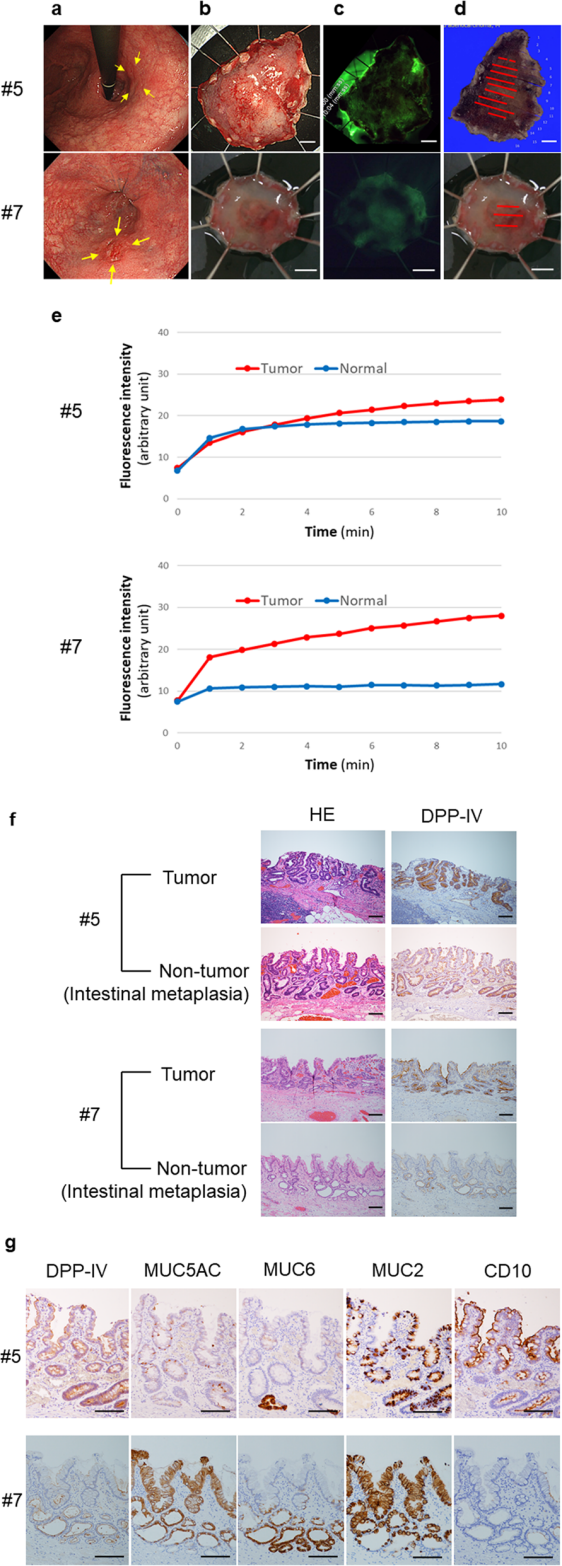
Fluorescence imaging with EP-HMRG of adenocarcinoma with a background of intestinal metaplasia located at the G (case #5) and E with LSBE (case #7). **a** Endoscopic imaging with white light before endoscopic submucosal dissection (ESD). **b** Endoscopic imaging with white light after ESD. **c** Fluorescence imaging after EP-HMRG spraying. **d** Resected specimen mapping for the tumor region. The adenocarcinoma is shown as red lines. **e** Time course of the fluorescence intensity of the tumor lesion and the non-tumor region after EP-HMRG spraying. **f** Hematoxylin and eosin staining and immunohistochemical examination investigating DPP-IV expression of the tumor lesion and non-tumor region. **g** Immunohistochemical examination investigating the subtype of intestinal metaplasia in the non-tumor region. A decrease in the expression of MUC5AC and MUC6 and the positive expression of MUC2 and CD10 indicate complete intestinal metaplasia (#5). The positive expression of MUC5AC, MUC6, and MUC2 and negative expression of CD10 indicates incomplete intestinal metaplasia (#7). Scale bars of (**b**–**d**), 5 mm. Scale bars of **(f** and **g**), 200 μm

In four cases, the tumor lesion developed with a background of intestinal metaplasia. Three of these four cases (cases #2, #5, and #11) had extensive complete intestinal metaplasia in almost all areas adjacent to the tumor lesion. In those cases, both the non-tumor region and the tumor lesion became fluorescent after spraying EP-HMRG (Fig. 3a–e, case #5). The pathological examination revealed that both the tumor lesion and the intestinal metaplasia expressed DPP-IV (Fig. 3f and g, case #5). However, the lesion in another case, which arose from LSBE (case #7), showed a sufficient CBR because the DPP-IV expression in the incomplete intestinal metaplasia was not as strong as a tumor lesion (Fig. 3a-g, case #7).

### Diagnostic performance of EP-HMRG for detecting early EGJ carcinoma

**Fig. 4:**
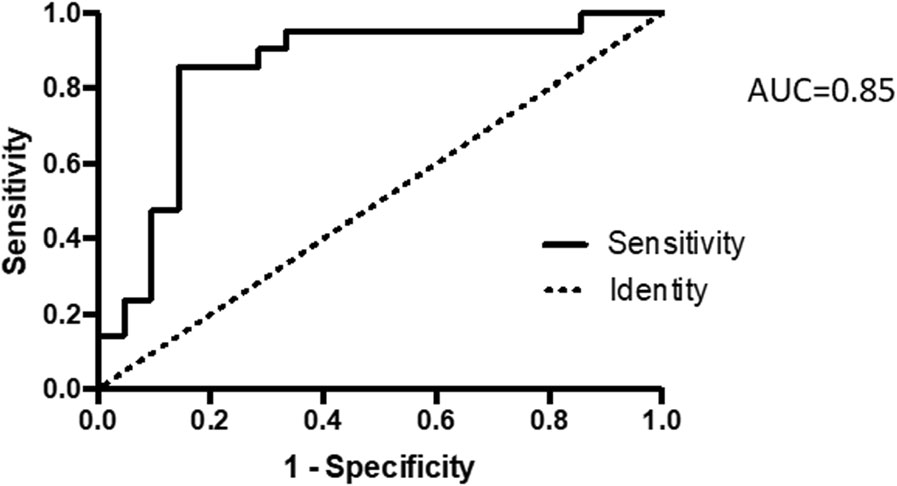
Receiver operating characteristic (ROC) curves of the EP-HMRG observations of all 21 cases for the detection of early adenocarcinoma of the esophagogastric junction

We performed a ROC analysis of the diagnostic performance of EP-HMRG for detecting early EGJ carcinoma. Including all 21 cases, the sensitivity, specificity, and accuracy values were 85.7, 85.7, and 85.7%, respectively (area under the curve [AUC] = 0.85; 95% confidence interval [CI] 0.72–0.98; Fig. 4); however, after excluding the cases with a background of intestinal metaplasia, those values were 88.2, 88.2, and 88.2%, respectively (AUC = 0.86; 95% CI 0.72–1.00). These results demonstrate the usefulness of EP-HMRG in clinical applications, especially for diagnosing lesions without a background of intestinal metaplasia.

## Discussion

In this study, we evaluated the usefulness of EP-HMRG for detecting early EGJ adenocarcinoma and found that the tumor lesion became fluorescent immediately after EP-HMRG spraying and had a sufficient CBR, especially in cases that developed without a background of intestinal metaplasia. To the best of our knowledge, we showed for the first time that EGJ adenocarcinoma can be detected by fluorescence molecular imaging.

Although recently developed modalities, such as NBI and magnifying endoscopy, have been developed for the detection and diagnosis of cancer, extensive endoscopist skills and experience are still necessary for an accurate diagnosis of EGJ adenocarcinoma, including Barrett’s adenocarcinoma. In particular, in cases with lesions that extend under the squamous epithelium, it is difficult to detect the lesions. In these cases, glandular spots composed of tumor glands that arise and open on the squamous epithelium are indicative of the existence of carcinoma and give us an opportunity to indicate existence of carcinoma. However, endoscopists often cannot recognize whether these glandular spots are tumor or columnar epithelial islands without atypia, which are found in the lower esophagus within 1 cm of the squamocolumnar junction in 57% of patients [28]. This is because most EGJ adenocarcinomas are histologically differentiated, and glandular spots composed of well-differentiated adenocarcinomas have similar surface patterns to columnar epithelial islands. When these spots are very small, endoscopists have difficulty determining whether the glandular spots open on the squamous epithelium are tumor tissue, even with new endoscopic modalities. In the present study, even exceptionally small glandular spots were made more readily visible by EP-HMRG. We confirmed that the fluorescence imaging with EP-HMRG enabled the visualization of such cancers that would otherwise be difficult to detect.

It has been reported that DPP-IV is strongly expressed at the brush border of the intestinal metaplasia as well as in gastric cancer [29]. In the present study, all three cases that were entirely surrounded by complete intestinal metaplasia that was characterized by a brush border did not show a sufficient CBR because both the tumor lesion and the intestinal metaplasia region expressed DPP-IV and therefore became fluorescent after EP-HMRG spraying. Most gastric cancers in Japan have various degrees of atrophy and intestinal metaplasia following *Helicobacter pylori*-associated gastritis in the background mucosa. On the other hand, the case of LSBE in the present study (case #7) with a background of incomplete metaplasia had low expression of DPP-IV; therefore, the CBR was relatively high (2.4). It has been reported that specialized columnar epithelium, which is regarded as a characteristic of Barrett’s mucosa in European and American countries, is incomplete intestinal metaplasia without a brush border [30]. Therefore, it is possible that Barrett’s adenocarcinoma could be detected by EP-HMRG with a good CBR. Further studies with a larger number of cases will be required to confirm this hypothesis.

Onoyama et al. reported that the EP-HMRG absorption and emission spectra depend on pH, while EP-HMRG itself becomes highly fluorescent in acidic conditions [18]. In case #14, the fluorescence intensity of the background was as high as that of the tumor lesion despite the lack of DPP-IV expression in the background (data not shown). This is the only case in which a pathological feature did not correspond to the CBR, and it appeared that EP-HMRG became fluorescent due to the presence of gastric acid.

In the present study, one patient developed a tumor after radiotherapy (case #4, Additional file 1: Figure S1); the tumor did not become fluorescent after spraying, and it did not express DPP-IV. Although the reason for this is not clear, we have previously reported in a study of head and neck squamous cell carcinoma that five tumors that developed after radiotherapy did not express DPP-IV and did not show sufficient CBR [19].

There are several limitations to this study. As our study was performed ex vivo, the DPP-IV activity could have potentially decreased following tumor resection. It took 10–20 min for the fluorescence imaging to initiate after tumor resection, and the imaging was performed at room temperature rather than at 37 °C, which is the condition we previously reported [19]. Therefore, we would expect that the EP-HMRG application would be more active in an in vivo clinical study than in an ex vivo study using resected specimens. Additionally, in cases with morphological 0–IIa or 0–I, fluorescence was not observed in the middle of the raised areas but was observed at the margin of these areas. There is a possibility that the probe flew out to the surroundings due to gravity before incorporated into the cancer cells. Therefore, it appears that an EP-HMRG suspension with high viscosity would resolve these concerns.

However, it is generally easy to detect elevated lesions with conventional endoscopy. Furthermore, most of the cases with elevated portions in this study coexisted with a 0–IIb or 0–IIc component, and fluorescence was observed in these areas.

## Conclusions

In conclusion, our data suggest that the topical spraying of EP-HMRG enabled rapid fluorescence imaging of early EGJ adenocarcinoma. Further studies to validate for cases with intestinal metaplasia and to evaluate the safety of this probe, as well as the development of fluorescence endoscopy to capture the fluorescence emitted by EP-HMRG, are necessary. We expect that many endoscopists will be able to more easily detect EGJ carcinoma at an early stage by applying EP-HMRG.

## Supplementary information

**Additional file 1: Supplementary Figure.** Fluorescence imaging with EP-HMRG and pathological examination of adenocarcinoma after radiotherapy (case #4). (**a**) Endoscopic imaging with white light before endoscopic submucosal dissection (ESD). Arrows indicate the tumor lesion. (**b**) Endoscopic imaging with white light after ESD. (**c**) Fluorescence imaging after EP-HMRG spraying. (**d**) Resected specimen mapping for the tumor region. The adenocarcinoma is shown as red lines. (**e**) Time course of the fluorescence intensity of the tumor lesion and the non-tumor region after EP-HMRG spraying. (**f**) Hematoxylin and eosin staining of the tumor lesion and the non-tumor region. (**g**) Immunohistochemical examination investigating DPP-IV expression in the tumor lesion and the non-tumor region. Scale bars of **b–d**, 5 mm. Scale bars of **f** and **g**, 200 μm.

## Data Availability

The datasets used and/or analyzed during the current study are available from the corresponding author on reasonable request.

## Abbreviations

CBR: Contrast-to-background ratio
DMSO: Dimethylsulfoxide
DPP: Dipeptidylpeptidase
EGJ: Esophagogastric junction
ESD: Endoscopic submucosal dissection
HMRG: Hydroxymethyl rhodamine green
PBS: Phosphate-buffered saline
ROI: Regions of interest
