# Supplementary figures and images for: Detection of early adenocarcinoma of the esophagogastric junction by spraying an enzyme-activatable fluorescent probe targeting Dipeptidyl peptidase-IV
Source: BMC Cancer. 2020 Jan 28;20:64. doi: 10.1186/s12885-020-6537-9 (PMC6988364; doi:10.1186/s12885-020-6537-9)

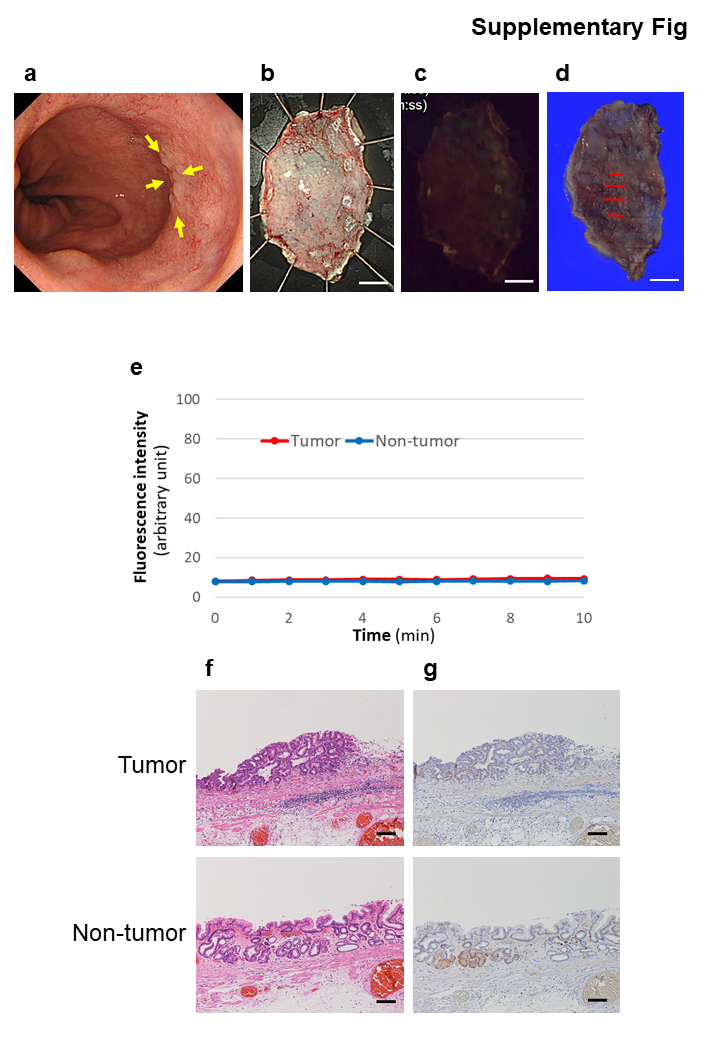

Supplement: Supplementary file 1 — Additional file 1: Supplementary Figure. Fluorescence imaging with EP-HMRG and pathological examination of adenocarcinoma after radiotherapy (case #4). (a) Endoscopic imaging with white light before endoscopic submucosal dissection (ESD). Arrows indicate the tumor lesion. (b) Endoscopic imaging with white light after ESD. (c) Fluorescence imaging after EP-HMRG spraying. (d) Resected specimen mapping for the tumor region. The adenocarcinoma is shown as red lines. (e) Time course of the fluorescence intensity of the tumor lesion and the non-tumor region after EP-HMRG spraying. (f) Hematoxylin and eosin staining of the tumor lesion and the non-tumor region. (g) Immunohistochemical examination investigating DPP-IV expression in the tumor lesion and the non-tumor region. Scale bars of b–d, 5 mm. Scale bars of f and g, 200 μm. [file 12885_2020_6537_MOESM1_ESM.tif]
